# Sex-specific tonic 2-arachidonoylglycerol signaling at inhibitory inputs onto dopamine neurons of Lister Hooded rats

**DOI:** 10.3389/fnint.2013.00093

**Published:** 2013-12-19

**Authors:** Miriam Melis, Marta De Felice, Salvatore Lecca, Liana Fattore, Marco Pistis

**Affiliations:** ^1^Division of Neuroscience and Clinical Pharmacology, Department of Biomedical Sciences, University of Cagliari, Cittadella Universitaria di MonserratoMonserrato, Italy; ^2^Institut du Fer à MoulinParis, France; ^3^Institute of Neuroscience, National Research Council, CagliariItaly

**Keywords:** addiction, cannabinoid, dopamine, electrophysiology, sex difference, synaptic plasticity, ventral tegmental area, 2-arachidonoylglycerol

## Abstract

Addiction as a psychiatric disorder involves interaction of inherited predispositions and environmental factors. Similarly to humans, laboratory animals self-administer addictive drugs, whose appetitive properties result from activation and suppression of brain reward and aversive pathways, respectively. The ventral tegmental area (VTA) where dopamine (DA) cells are located is a key component of brain reward circuitry, whereas the rostromedial tegmental nucleus (RMTg) critically regulates aversive behaviors. Reduced responses to either aversive intrinsic components of addictive drugs or to negative consequences of compulsive drug taking might contribute to vulnerability to addiction. In this regard, female Lister Hooded (LH) rats are more vulnerable than male counterparts to cannabinoid self-administration. We, therefore, took advantage of sex differences displayed by LH rats, and studied VTA DA neuronal properties to unveil functional differences. Electrophysiological properties of DA cells were examined performing either single cell extracellular recordings in anesthetized rats or whole-cell patch-clamp recordings in slices. *In vivo*, DA cell spontaneous activity was similar, though sex differences were observed in RMTg-induced inhibition of DA neurons. *In vitro*, DA cells showed similar intrinsic and synaptic properties. However, females displayed larger depolarization-induced suppression of inhibition (DSI) than male LH rats. DSI, an endocannabinoid-mediated form of short term plasticity, was mediated by 2-arachidonoylglycerol (2-AG) activating type 1-cannabinoid (CB1) receptors. We found that sex-dependent differences in DSI magnitude were not ascribed to CB1 number and/or function, but rather to a tonic 2-AG signaling. We suggest that sex specific tonic 2-AG signaling might contribute to regulate responses to aversive intrinsic properties to cannabinoids, thus resulting in faster acquisition/initiation of cannabinoid taking and, eventually, in progression to addiction.

## INTRODUCTION

Drug addiction is defined as a chronic relapsing brain disease characterized by compulsive drug seeking and taking, despite negative consequences. Similarly to other psychiatric disorders, drug addiction depends on interaction of inherited predispositions and environmental factors. Among these, gender differences have been reported during all phases of addiction cycle (Becker and Hu, 2008 and references therein), which includes initiation/acquisition, escalation and progression to addiction, with subsequent withdrawal followed by relapse (Koob and Kreek, 2007).

Addictive drugs share the property of being self-administered by laboratory animals, and acquisition of drug taking behavior depends on several variables including aversive characteristics intrinsic to many addictive drugs (Stolerman, 1992). Indeed, aversive properties of drugs of abuse, such as cannabinoids and alcohol, play a key role in acquisition phases of self-administration in laboratory animals (Quinn et al., 2008; Rezvani et al., 2010). In addition, vulnerability to drug abuse and dependence might also result from diminished responses to harmful consequences of compulsive drug taking (Riley, 2011).

Addictive drugs also share the property of activating the brain reward circuitry, which stems from the ventral tegmental area (VTA) where dopamine (DA) cells are located (Kalivas et al., 2006). Caudally located to the VTA, the rostromedial tegmental nucleus (RMTg) is a GABAergic structure sending inhibitory projections to VTA DA neurons, and serving as a major brake for these cells (Jhou et al., 2009b). The RMTg plays a role in aversive behaviors, particularly those involving behavioral inhibition (Jhou et al., 2009a). Hence, VTA is ideally located as a possible hub between aversion- and reward-responding brain regions (Barrot et al., 2012), where DA neuronal activity contributes not only to the predictive validity of information but also to learning about rewards and punishments (Bromberg-Martin and Hikosaka, 2009; Matsumoto and Hikosaka, 2009).

Dopamine neuronal activity results by delicate balance between both intrinsic and extrinsic mechanisms (Schmitz et al., 2003; Brown et al., 2009). Endocannabinoids, retrograde signaling lipid molecules, play an important role in selectively tuning synapses impinging upon DA cells, and may act as a switch by selectively regulating a given synapse, and ultimately adjusting DA neuron output (Melis and Pistis, 2007, 2012). Particularly, given the strong GABA background input arising from diverse districts (i.e., ventral pallidum, nucleus accumbens, RMTg, VTA), endocannabinoids – by silencing one or more inhibitory inputs- might sensitize DA cells towards excitatory inputs evoked by rewarding stimuli, such as those associated with drugs of abuse (Melis and Pistis, 2012; Melis et al., 2012). This might result into enhanced impulse activity of DA cells and DA release in terminal regions. These latter are involved in the onset of drug addiction, as well as in the susceptibility of relapse (Marinelli et al., 2003). Additionally, individual vulnerability to drug seeking behavior is associated with increased impulse activity of DA cells, whereas a decreased DA cell activity is linked with resistance to the development of addictive behaviors (Marinelli and White, 2000; Brandon et al., 2001; Marinelli et al., 2003, 2006; Melis et al., 2009). Accordingly, sexual dimorphisms in DA release and uptake, and in DA responses to psychostimulants have long been reported (Schneider and Norton, 1979; Savageau and Beatty, 1981; Becker et al., 1982; Camp et al., 1986; van Haaren and Meyer, 1991; Haney et al., 1994; Walker et al., 2000, 2001, 2006). Sex is also an important modulating factor in a variety of cannabinoid effects on behavior, being females usually more sensitive than males to cannabinoids (Craft, 2005; Fattore and Fratta, 2010). Accordingly, female Lister Hooded (LH) rats displayed vulnerability to cannabinoid self-administration (Fattore et al., 2007, 2009, 2010). Particularly, female LH rats acquire faster, maintain higher and extinguish later cannabinoid self-administration when compared to their male counterparts. Notably, these sex-dependent differences appear to rely, at least in part, on the presence of ovarian hormones (Fattore et al., 2007, 2010). Indeed, we reported that ovary ablation typically dampens operant behaviors, from acquisition of cannabinoid self-administration to reinstatement of cannabinoid-seeking behavior (Fattore et al., 2007, 2010). Thus, performance of ovariectomized female LH rats resulted more similar to that of males rather than to cycling females (Fattore et al., 2009). Similarly, estradiol-dependent differences in behaviors associated with greater vulnerability to drug addiction have been recently described in female LH rats (Castelli et al., 2013).

Among potential mechanisms underlying neurobiological basis of sex dimorphism in cannabinoid effects on behavior (Fattore, 2012), several key pieces of information on DA functioning are yet unknown. Primarily, is VTA DA cell spontaneous activity different between sexes? Are there any sex differences in properties of afferent synapses onto VTA DA cells? Are endocannabinoids and CB1 receptors involved in sex-dependent predisposition to acquire cannabinoid self-administration? Thus, we examined the electrophysiological properties of VTA DA neurons of female and male LH rats, both *in vivo* and *in vitro*, in order to elucidate the neurobiological basis of their sex-dependent behavioral responsiveness to cannabinoids.

## MATERIALS AND METHODS

### ANIMALS

All procedures were performed in accordance with the Guidelines for the Care and Use of Mammals in Neuroscience and Behavioral Research (National Research Council 2004) and EEC Council Directive (219/1990 and 220/1990). We made all efforts to minimize pain and suffering, and to reduce the number of animals used. Animals were housed in groups of three to six in standard conditions of temperature (21 ± 1°C) and humidity (60%) under a 12 h/12 h light/dark cycle (lights on at 7.00 am) with food and water available *ad libitum.* For the *in vivo* electrophysiological experiments female rats were housed together for a period of nearly 75 days. Since the ovarian cycle of female rats become synchronized when they live together, as does the cycle of many other mammals (Schank and McClintock, 1992), we can assume that their estrous cycle is synchronized. For the *in vitro* electrophysiological experiments the age of the animals used is considered as a period before the onset of puberty and, therefore, any difference between sexes should be ascribed to organizational rather than activational effects of sex hormones.

### *IN VIVO* ELECTROPHYSIOLOGY

Male and female Lister Hooded rats (250–350 g, Harlan, Italy) were anesthetized with urethane (1.3 g/kg, i.p.), their femoral vein was cannulated for i.v. administration of pharmacological agents and they were placed in the stereotaxic apparatus (Kopf, Tujunga, CA, USA) with their body temperature maintained at 37 ± 1°C by a heating pad. Thereafter, the scalp was retracted, and one burr hole was drilled above the VTA (AP, -5.4 to -5.8 mm from bregma; L, 0.4–0.6 mm from midline) for the placement of a recording electrode. Single unit activity of neurons located in VTA (V, 7.0–8.0 mm from the cortical surface) was recorded extracellularly with glass micropipettes filled with 2% pontamine sky blue dissolved in 0.5 M sodium acetate (impedance, 2–5 MΩ). Single unit activity was filtered (bandpass, 0.1–10000 Hz) and individual spikes were isolated by means of a window discriminator (Digitimer, Hertfordshire, UK), displayed on a digital storage oscilloscope (TDS 3012, Tektronics, Marlow, UK). Experiments were sampled on line and off line with Spike2 software (Cambridge Electronic Design, Cambridge, UK) by a computer connected to CED 1401 interface (Cambridge Electronic Design, Cambridge, UK). Single units were isolated and identified according to the already published criteria (Lecca et al., 2012). VTA DA neurons were selected when all criteria for identification were fulfilled: firing rate <10 Hz; duration of action potential >2.5 ms as measured from start to end. Bursts were defined as the occurrence of two spikes at interspike interval <80 ms, and terminated when the interspike interval exceeded 160 ms (Grace and Bunney, 1983). Isolated DA neurons were recorded for 2–3 min to establish basal firing properties. To evaluate the inhibitory input arising from the RMTg to the VTA, a Formvar-coated stimulating stainless steel bipolar electrode (250 μm tip diameter) was aimed at the ipsilateral RMTg (AP, -7.2 mm from bregma; L, 0.8 mm from midline; V, 7 mm from cortical surface and with an inclination of 20° anteroposterior on the coronal plane) according to the stereotaxic atlas of Paxinos and Watson (2007). The stimulation protocol was essentially as described previously (Lecca et al., 2011). Briefly, once a cell was selected, electrical stimuli consisting of single, monophasic, rectangular pulses (0.5 mA, 0.3 ms) were delivered to the RMTg at 1 Hz. Responses to electrical stimulation of the RMTg were evaluated, and a peristimulus time histogram (PSTH) was generated on-line for each neuron. PSTHs were built from 100 stimuli and displayed using 1 ms bin width. A cell was considered inhibited or excited when the number of action potentials/bin (bin length = 1 ms) in the 50 ms after the stimulus was significantly lower or higher (one-way ANOVA for repeated measures), respectively, than baseline levels (the number of action potentials/bin in the 50 ms period before the stimulus). The duration of stimulus-evoked inhibition was defined as the time of complete cessation of firing after the stimulus (Lecca et al., 2012). WIN 55,212-2 was dissolved in Tween 80 (1%), then sonicated and diluted in saline (1ml/kg).

### *IN VITRO* ELECTROPHYSIOLOGY

The preparation of VTA slices was as described previously (Melis et al., 2009, 2013). Briefly, male and female Lister Hooded rats (12–25 days) were anesthetized with halothane and euthanized. A block of tissue containing the midbrain was rapidly dissected and sliced in the horizontal plane (300 μm) with a vibratome (Leica) in ice-cold low-Ca^2^^+^ solution containing (in mM): 126 NaCl, 1.6 KCl, 1.2 NaH_2_PO_4_, 1.2 MgCl_2_, 0.625 CaCl_2_, 18 NaHCO_3_, and 11 glucose). Slices were transferred to a holding chamber with artificial cerebrospinal fluid (ACSF, 37°C) saturated with 95% O_2_ and 5% CO_2_ containing (in mM): 126 NaCl, 1.6 KCl, 1.2 NaH_2_PO_4_, 1.2 MgCl_2_, 2.4 CaCl_2_, 18 NaHCO_3_, and 11 glucose. Slices were allowed to recover for at least 1 h before being placed, as hemislices, in the recording chamber and superfused with the ACSF (34–36°C) saturated with 95% O_2_ and 5% CO_2_. Cells were visualized with an upright microscope with infrared illumination (Axioskop FS 2 plus, Zeiss), and whole-cell patch clamp recordings were made by using an Axopatch 200B amplifier (Axon Instruments, CA, USA). Current-clamp experiments and IPSCs recordings were made with electrodes filled with a solution containing the following (in mM): 144 KCl, 10 HEPES, 3.45 BAPTA, 1 CaCl_2_, 2.5 Mg_2_ATP, and 0.25 Mg_2_GTP (pH 7.2–7.4, 275–285 mOsm). All EPSC recordings were made with electrodes filled with a solution containing the following (in mM): 117 Cs methansulfonic acid, 20 HEPES, 0.4 EGTA, 2.8 NaCl, 5 TEA-Cl, 2.5 Mg_2_ATP, and 0.25 Mg_2_GTP (pH 7.2–7.4, 275–285 mOsm). Experiments were begun only after series resistance had stabilized (typically 15–40 MΩ). Series and input resistance were monitored continuously on-line with a 5 mV depolarizing step (25 ms). Data were filtered at 2 kHz, digitized at 10 kHz, and collected on-line with acquisition software (pClamp 8.2, Axon Instruments, CA, USA). DA neurons from the posterior VTA were identified by the presence of a large *I*_h_ current (>128 pA; Johnson and North, 1992) that was assayed immediately after break-in, using a series of incremental 10 mV hyperpolarizing steps from a holding potential of -70 mV. DA neurons were identified according to the already published criteria (Melis et al., 2007, 2008, 2013): cell morphology and anatomical location (i.e., medial to the medial terminal nucleus of the accessory optic tract), large hyperpolarization activated current (I_h_ > 100 pA), slow pacemaker-like firing rate (<5 Hz), long action potential duration (>2 ms). A bipolar stainless steel stimulating electrode (FHC, USA) was placed either 100 μm rostral or 500 μm caudal to the recording electrode and was used to stimulate at a frequency of 0.1 Hz. Paired stimuli were given with an interstimulus interval of 50 ms, and the ratio between the second and the first PSCs was calculated and averaged for a 5 min baseline. The depolarizing pulse used to evoke depolarization-induced suppression of inhibition (DSI) was a 500 ms to 5 s step to +40mV from holding potential (-70 mV), as previously reported (Melis et al., 2004, 2009). The magnitude of DSI was measured as percentage of the mean amplitude of consecutive IPSCs after depolarization (acquired between 5 and 15 s after the end of the pulse) relative to that of five IPSCs before the depolarization (Melis et al., 2004). All the numerical data are given as mean ± SEM and compared using the Student’s *t*-test. Each slice received only a single drug exposure. Drugs were applied in known concentrations to the superfusion medium. All the drugs were dissolved in DMSO. The final concentration of DMSO was <0.01 %. Averaged data from different experiments are presented as mean ± SEM. Bath application of WIN was performed as follows: WIN was applied for 5 min at the lowest concentration, then, another 5 min with the next increasing WIN concentration. The effect of WIN on GABA_A_ IPSCs was taken at the 5th minute of bath application and normalized to the baseline (5 min before drug application). We chose this protocol because it has been shown that at physiological temperatures WIN-induced effects on GABA_A_ IPSCs recorded from VTA DA neurons reached its maximum at this time (Lecca et al., 2012).

### STATISTICAL ANALYSIS

All the numerical data are given as mean ± S.E.M. Data were analyzed by utilizing two-way ANOVA for repeated measures (treatment × time), or one-way ANOVA or Student’s *t*-test for repeated measures, when appropriate. *Post hoc* multiple comparisons were made using either the Dunnett’s test or Bonferroni’s test. The significance level was established at *P* < 0.05.

## RESULTS

### VTA DA NEURONAL ACTIVITY OF FEMALE AND MALE LH RATS *IN VIVO*

**FIGURE 1:**
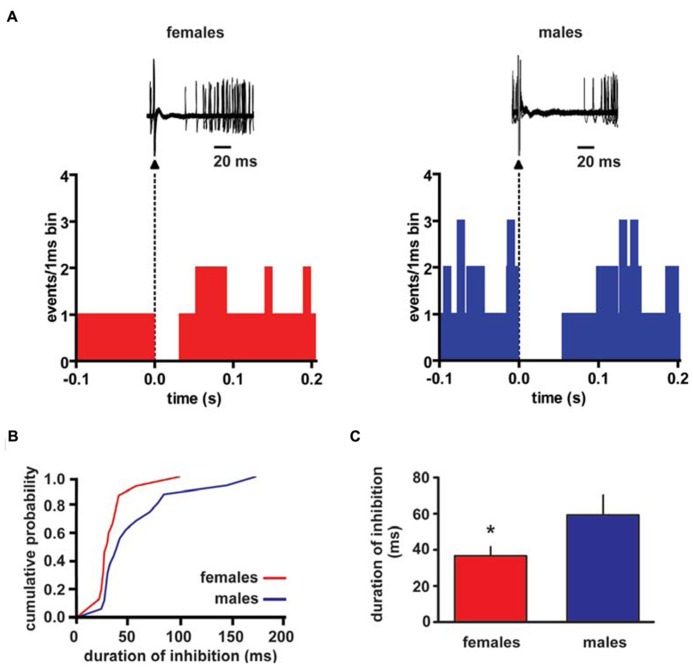
**Rostromedial tegmental nucleus (RMTg) stimulation induces a shorter inhibition of VTA DA neuron spontaneous activity in female LH rats.** **(A)** The top panel shows traces acquired from a digital oscilloscope representing typical examples of a VTA DA neuron recorded in female (left panel) and male (right panel) LH rat, whose stimulation from RMTg was evoked by delivering rectangular current pulses (1 Hz, 0.5 mA, 0.3 ms). RMTg-stimulation produced a full suppression in firing activity of VTA DA neurons for ~36 and ~60 ms in female and male LH rats, respectively. The arrowheads indicate the stimulus artifacts. The bottom panel shows peristimulus time histograms of the cells displayed in the top panel (100 consecutive sweeps). **(B)** Cumulative distribution plot of duration of inhibition from RMTg shows a reduced RMTg-evoked inhibition of VTA DA cells in female (*n* = 15) and male (*n* = 16) rats. **(C)** Summary graph histogram showing sex differences in averaged effect induced by RMTg stimulation on VTA DA cell activity. Data are expressed as mean ± SEM. *P < 0.05.

**FIGURE 2:**
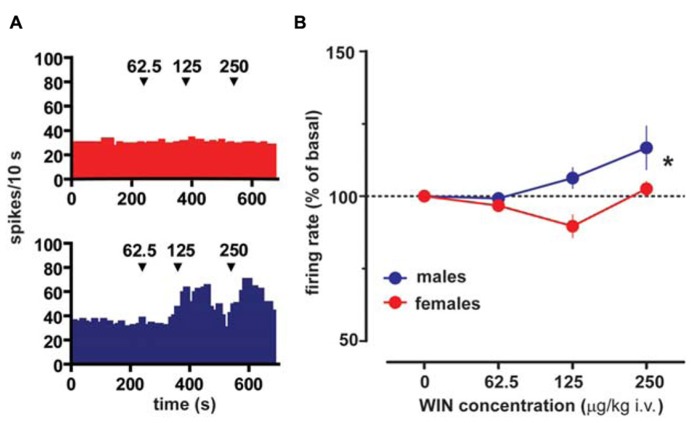
**Sex specific effect of the cannabinoid receptor agonist WIN55212-2 on VTA DA neuron firing rate.** **(A)** Effect produced by cumulative doses of WIN on a VTA DA neuron recorded from a female (top) and a male (bottom) LH rat. Arrows indicate the time of injection. Numbers above arrows indicate the dosages expressed in μg/kg i.v. Note the lack of stimulating effect of WIN in a female LH rat VTA DA neuron. **(B)** Dose–response curves depicting the sex specific stimulating effect of cumulative doses of WIN (*n* = 8 for both sexes) on the firing rate of VTA DA neurons in LH rats. Data (mean ± SEM) are expressed as percentages of basal firing rate. *P < 0.05 with respect to pre-drug level (two-way ANOVA followed by Dunnett’s test).

Extracellular single unit recordings were made from female (*n* = 5) and male (*n* = 5) LH rat posterior VTA DA neurons. We chose this location within the VTA because most of the nucleus accumbens-projecting DA neurons are mainly located in this quadrant of the VTA (Ford et al., 2006; Lammel et al., 2012). Only DA cells meeting the electrophysiological criteria for DA neurons were included in this study. Action potentials recorded from both female and male LH rats displayed a typical broad notched-waveform. Since female LH rats were housed together their estrous cycle was synchronized. However, we chose to perform the recordings randomly at different phases of the cycle to rule out any activational effects of fluctuating hormonal levels. An analysis of the number of spontaneously active VTA DA neurons in female and male LH rats did not reveal any difference, since the mean (±S.E.M.) number of DA neurons encountered in the VTA was 1.6 ± 0.2 and 1.6 ± 0.3 cells/track, respectively (**Table 1**). Additionally, the averaged spontaneous firing rate of VTA DA cells of female and male LH rats was not different, being 2.6 ± 0.2 (*n* = 69) and 3.0 ± 0.2 Hz (*n* = 60), respectively (**Table 1**; *t*-test: *t* = 1.50; *P* > 0.05). Similarly, no difference in other standard basal electrophysiological parameters was found between female and male LH rats (**Table 1**). In another group of animals (*n* = 6 and 8 male and female rats, respectively), after 2 min of stable recording of firing rate, we tested their response to RMTg stimulation (1 Hz, 0.5 mA, 0.3 ms). The electrical stimulation of the RMTg induced an inhibition of spontaneous activity in 25% (15 out of 60) and 30% (16 out of 54) of the neurons examined in female and male LH rats, respectively. Representative oscilloscope traces illustrating the typical RMTg-induced suppression of VTA DA neuronal discharge are shown in **Figure 1A**. Noteworthy, the duration of inhibition evoked by RMTg stimulation was shorter in female (36.4 ± 5.2 ms, *n* = 15) than male (59.3 ± 10.9 ms, *n* = 16) LH rats (Mann-Whitney test, *P* < 0.05, **Figure 1**). Additionally, stimulating properties on VTA DA cell firing rate of CB1 receptor agonist WIN 55,212-2 (WIN, from 62.5 up to 250 μg/kg i.v. in a cumulative dose regimen; French et al., 1997; Gessa et al., 1998) were blunted in female LH rats (two-way ANOVA, *F*_1,42_ = 7.57, *P* < 0.05, *n* = 8 for both sexes; **Figure 2)**.

**Table 1:** Electrophysiological properties of VTA dopamine neurons recorded from male and female LH rats *in vivo*

	Males	Females	
Frequency (Hz)	3.00 ± 0.19 (*n* = 60)	2.62 ± 0.17 (*n* = 69)	NS
Percentage of spikes in burst	7.50 ± 1.84 (*n* = 60)	11.41 ± 2.01 (*n* = 69)	NS
Spikes/burst	72.53 ± 21.26 (*n* = 36)	75.98 ± 16.25 (*n* = 50)	NS
Mean spikes in burst	2.52 ± 0.11 (*n* = 36)	2.59 ± 0.15 (*n* = 50)	NS
Burst rate (Hz)	0.16 ± 0.04 (*n* = 36)	0.20 ± 0.05 (*n* = 50)	NS
Mean intraburst frequency (Hz)	41.53 ± 6.80 (*n* = 36)	35.55 ± 2.12 (*n* = 50)	NS
Mean burst duration (ms)	105.1 ± 11.36 (*n* = 36)	106.2 ± 13.90 (*n* = 50)	NS
Cells per track (*n* rats)	1.58 ± 0.25 (*n* = 5)	1.58 ± 0.23 (*n* = 5)	NS

Taken together, these results suggest that while basal spontaneous activity of VTA DA cells show no sex differences, their response to CB1 receptor activation as well as to RMTg stimulation is differently regulated in female and male LH rats.

### COMPARISON OF VTA DA NEURONS FROM MALE AND FEMALE LH RATS *IN VITRO*

Whole-cell patch-clamp recordings were made from lateral posterior VTA DA neurons in rat horizontal slices containing the midbrain. VTA DA cells of female and male rats displayed similar electrophysiological characteristics which, apart from their location in the slice, facilitated their identification (Grace and Onn, 1989). Membrane properties of VTA DA cells were assessed immediately after break-in to avoid intracellular dialysis of the cell over time (Schaap et al., 1999). An analysis of the spontaneous activity of VTA DA cells did not reveal any difference between male and female LH rats. In particular, VTA DA cells fired spontaneously at a regular rate (females: 2.2 ± 0.6 Hz, *n* = 23; males: 2.6 ± 0.9 Hz, *n* = 23; Mann Whitney test, *P* > 0.05; data not shown), with long duration action potentials. The present *in vivo* results (**Figure 1**) revealed sex differences in afferent input control of spontaneous activity of VTA DA neurons. Therefore, to investigate whether changes at synaptic level might underlie sex differences observed in their drug taking behavior, we studied postsynaptic excitatory and inhibitory -mediated currents (i.e., EPSCs and IPSCs). Because an important factor controlling VTA DA cell firing is glutamatergic input from cortical and subcortical regions (Geisler et al., 2007), which forms excitatory synapses on VTA DA cells (Christie et al., 1985; Sesack and Pickel, 1992), in the first set of experiments we compared the property of excitatory synapses on VTA DA cells of female and male LH rats. The change in synaptic strength elicited by paired stimuli given at an interval of 50 ms was similar in female and male LH rats. In fact, excitatory synapses on VTA DA cells of both groups exhibited a similar paired-pulse facilitation (females: s2/s1 ratio = 1.6 ± 0.1; males: 1.8 ± 0.2; *n* = 7 for both sexes; two-tailed unpaired *t*-test, *P* > 0.05; data not shown). Similarly, when GABAergic synapses were examined by stimulating either rostral (about 150 μm from the recording electrode) or caudal (about 550 μm from recording electrode) afferents no differences were found between male and female LH rats. In fact, there were no sex differences when rostral (females: s2/s1 ratio = 1.0 ± 0.1; males: 0.9 ± 0.1; *n* = 11 for both sexes; two-tailed Mann Whitney test, *P* > 0.5; data not shown) and caudal (females: s2/s1 ratio = 1.3 ± 0.1; males: 1.0 ± 0.1; *n* = 9 for both sexes; two-tailed Mann Whitney test, *P* > 0.5; data not shown) paired-pulse ratio were compared in female and male LH rats. Noteworthy, a statistical analysis revealed a difference between rostral and caudal paired-pulse ratio in female (unpaired two-tailed *t*-test, *P* < 0.05) but not male (unpaired two-tailed *t*-test, *P* > 0.5) LH rats. Altogether, these results suggest that VTA DA cell female and male LH rats display similar intrinsic and synaptic properties.

### DIFFERENCE IN ENDOCANNABINOID-MEDIATED TRANSMISSION BETWEEN FEMALE AND MALE LH RATS

Converging evidence suggests a bi-directional control between gonadal hormones and cannabinoids in the regulation of motivational processes (Lopez, 2010). Therefore, to investigate whether or not endocannabinoid system function was different between sexes, we compared an endocannabinoid-mediated form of short term plasticity occurring at both excitatory and inhibitory synapses, which is depolarization-induced suppression of excitation and inhibition, respectively (i.e., DSE/DSI; Diana and Marty, 2004), in LH rats. This original mode of action for endocannabinoids allows their retrograde signal to decrease neurotransmitter release via activation of CB1 receptors, and to regulate synaptic transmission in many brain regions (Lovinger, 2008). Thus, DSE/DSI represent a largely applied electrophysiological protocol to examine endocannabinoid nature and integrity at a given synapse. DSE expressed by VTA DA cells displayed no sex-dependent difference (two-way ANOVA, *F*_1,24_ = 2.44, *n* = 7 for both sexes; *P* > 0.05; data not shown).

**FIGURE 3:**
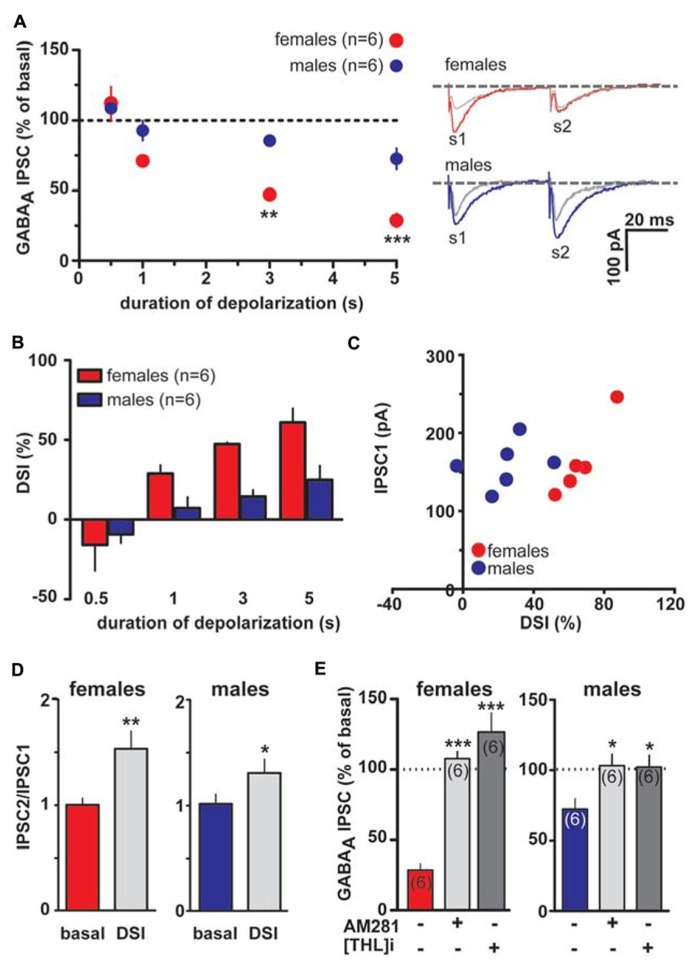
**Endocannabinoid modulation of inhibitory transmission arising from rostral afferents is reduced in male LH rats.** **(A)** The relationship between the depolarizing pulse duration and the relative amplitude of GABA_A_ IPSCs obtained after 5–15 s after the end of depolarization is plotted (left panel). GABA_A_ IPSCs amplitude was normalized to the averaged value (dotted line) before depolarization. Each symbol represents the averaged value obtained from different cells. Representative traces before and after DSI (5 s) are overlaid and shown (right panel). Gray traces represent the GABA_A_ IPSCs after the depolarizing step. **(B)** Averaged data for DSI induced by depolarizing pulses with a duration of 0.5, 1, 3, and 5 s are plotted. **(C)** No correlation was found between DSI magnitude and size of GABA_A_ IPSC in either sex. **(D)** Bar graphs summarizing the average paired-pulse ratio (IPSC2/IPSC1) of rostral GABA_A_ IPSCs for all cells recorded in male and female LH rats. No sex dimorphism was found in DSI-induced increased paired-pulsed ratio. **(E)** In the presence of either CB1 receptor antagonist (AM281) or inhibitor of *sn*-1-DAGL (THL) DSI could not be induced in either sex. Data are expressed as mean ± SEM. *P < 0.05, **P < 0.01, and ***P < 0.0001.

However, as shown in **Figures 3A,B** **1, 3** and **5** s depolarization induced a significant DSI at rostral synapses (rDSI) in female LH rats (IPSCs amplitude after the depolarizing pulse was 71.3 ± 5.3, 52.7 ± 1.1 and 39.0 ± 8.5% of baseline for 1, 3 and 5 s, respectively; *n* = 6; one-way ANOVA, *F*_4,24_ = 31.7, *P* < 0.0001). Remarkably, rDSI was dramatically larger in female LH rats when compared with the one induced in male LH rats (two-way ANOVA, *F*_1,24_ = 25.22, *P* < 0.0001; **Figures 3A,B**). Importantly, different magnitude of rDSI did not depend on the size of the first GABA_A_ IPSC (IPSC1; **Figure 3C**), being similar between female and male LH rats. rDSI was accompanied by an increased paired-pulse ratio (females, s2/s1 ratio from 1.1 ± 0.1 to 1.5 ± 0.2, paired *t*-test *t* = 3.42, *P* < 0.01; males, s2/s1 ratio from 1.0 ± 0.1 to 1.3 ± 0.1, paired *t*-test *t* = 2.76, *P* < 0.05, *n* = 6 for both sexes; **Figure 3D**) suggestive of a presynaptic locus of action. Additionally, as shown in **Figure 3E**, rDSI in both sexes required activation of CB1 receptors by 2-arachidonoylglycerol (2-AG), given that rDSI was absent in the presence of either CB1 receptor antagonist AM281 (0.5 μM; *t*-test, *P* < 0.05 and 0.0001 for males and females, respectively) or the inhibitor of *sn*-1-diacylglycerol lipase (*sn*-1- DAGL; Bisogno et al., 2003; i.e., 2-AG rate limiting synthesizing enzyme) THL (0.5 μM in the patch pipette, *t*-test, *P* < 0.05 and 0.001 for males and females, respectively).

**FIGURE 4:**
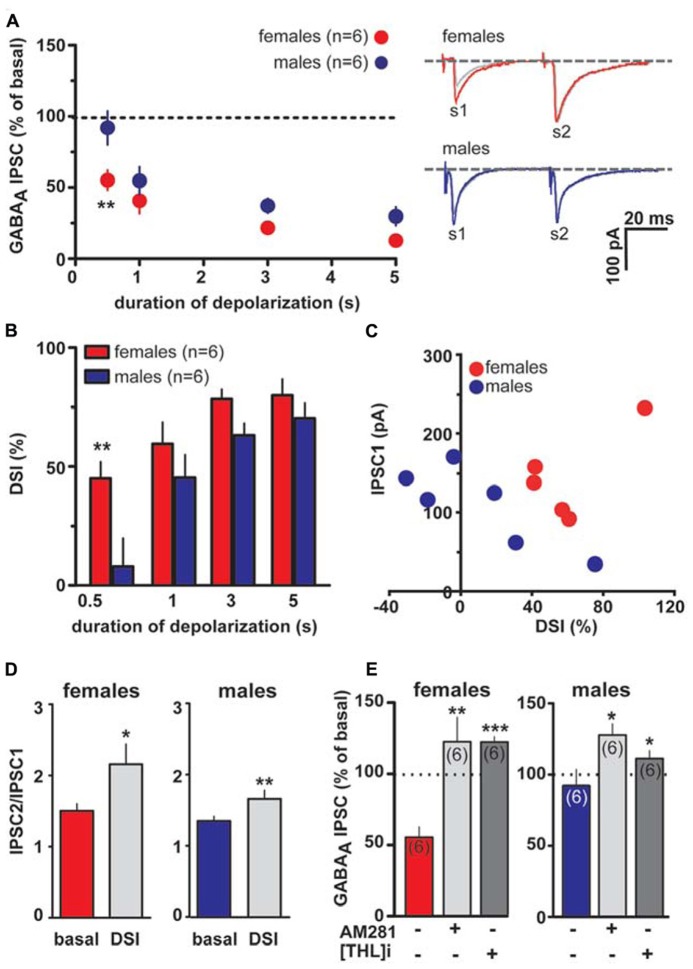
**Endocannabinoid modulation of inhibitory transmission arising from caudal afferents is reduced in male LH rats.** **(A)** The relationship between the depolarizing pulse duration and the relative amplitude of GABA_A_ IPSCs obtained after 5–15 s after the end of depolarization is plotted (left panel). GABA_A_ IPSCs amplitude was normalized to the averaged value (dotted line) before depolarization. Each symbol represents the averaged value obtained from different cells. Representative traces before and after DSI (0.5 s) are overlaid and shown (right panel). Gray traces represent the GABA_A_ IPSCs after the depolarizing step. **(B)** Averaged data for DSI induced by depolarizing pulses with a duration of 0.5, 1, 3, and 5 s are plotted. **(C)** No correlation was found between DSI magnitude and size of GABA_A_ IPSC in either sex. **(D)** Bar graphs summarizing the average paired-pulse ratio (IPSC2/IPSC1) of caudal GABA_A_ IPSCs for all cells recorded in male and female LH rats. No sex dimorphism was found in DSI-induced increased paired-pulsed ratio. **(E)** In the presence of either AM281 or THL DSI could not be induced in either sex. Data are expressed as mean ± SEM. *P < 0.05, **P < 0.01.

As shown in **Figures 4A,B**, DSI can be expressed by VTA DA neurons also when caudal afferents are stimulated (cDSI). Interestingly, female LH rats expressed cDSI at the shortest duration of depolarization (i.e., 0.5 s, IPSCs amplitude after the depolarizing pulse was 37.0 ± 10.0 and 108.5 ± 5.6% of baseline in females and males, respectively; *n* = 6; two-way ANOVA, *F*_1,20_ = 1.03, *P* < 0.001). Noteworthy, different magnitude of cDSI did not depend on the size of the first GABA_A_ IPSC (IPSC1; **Figure 4C**), being similar between female and male LH rats. Additionally, cDSI was accompanied by an increase in the paired-pulse ratio (females, s2/s1 ratio from 1.5 ± 0.1 to 2.1 ± 0.3, paired *t*-test *t* = 2.42, *P* < 0.05; males, s2/s1 ratio from 1.3 ± 0.1 to 1.7 ± 0.1, paired *t*-test *t* = 4.6, *P* < 0.01, *n* = 6 for both sexes; **Figure 4D**), suggestive of a presynaptic locus of action. Lastly, as shown in **Figure 4E**, cDSI in both sexes required activation of CB1 receptors by 2-AG, given that they were absent in the presence of either AM281 (0.5 μM; *t*-test, *P* < 0.05 and 0.01 for males and females, respectively) or THL (0.5 μM in the patch pipette, *t*-test, *P* < 0.05 and 0.001 for males and females, respectively).

**FIGURE 5:**
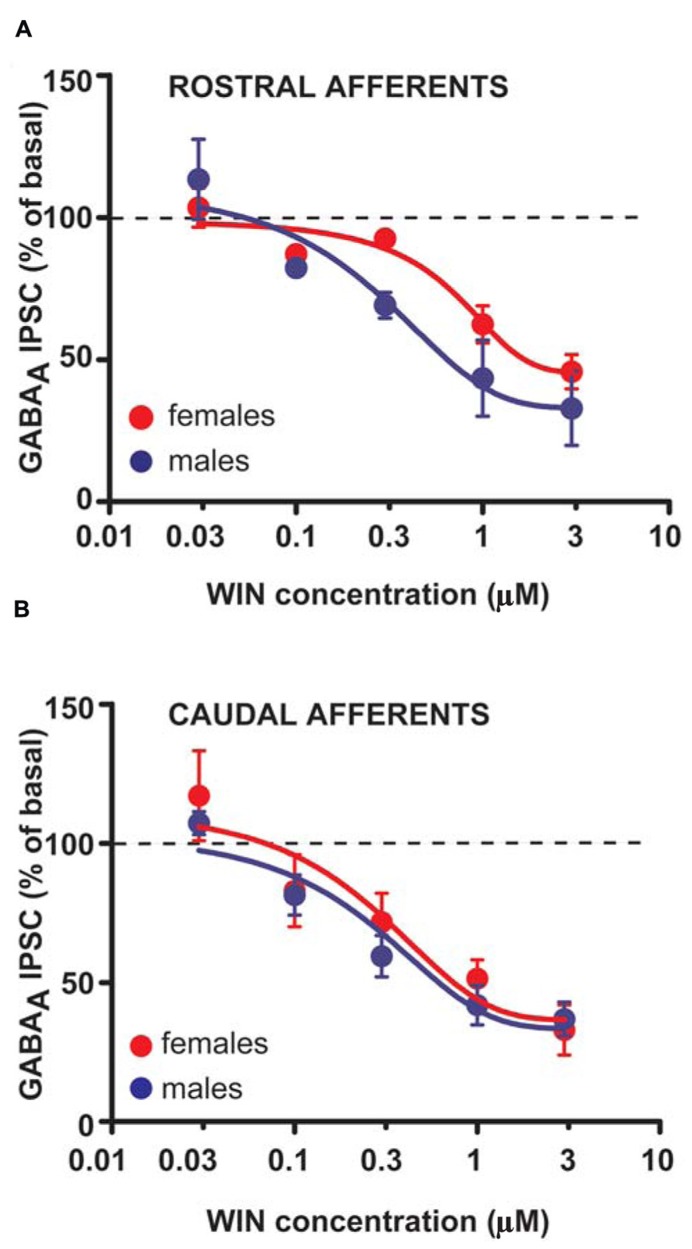
**CB1 receptor activation produces similar effects in male and female LH rats.** Dose–response curves for percentage inhibition in GABA_A_ IPSC amplitude, which were recorded from VTA DA cells and evoked by stimulating either rostral **(A)** (*n* = 5 for both sexes) or caudal **(B)** (*n* = 5 for both sexes) afferents, produced by the CB1 receptor agonist WIN. Each symbol represents the averaged value ( ± SEM) obtained from different cells.

The larger DSI expressed by VTA DA cells in female LH rats might reflect either higher levels of 2-AG, and/or an increased number or function of CB1 receptors. To assess whether differences in CB1 receptor function/number occur at GABAergic synapses arising from both rostral and caudal afferents onto VTA DA neurons of LH rats, WIN was applied in a separate group of experiments. Bath application of WIN (0.003–3 μM, 5 min) significantly reduced both rostral (*n* = 5; two-way ANOVA, *F*_4,32_ = 34.48, *P* < 0.0001; **Figure 5A**) and caudal GABA_A_ IPSCs (*n* = 5; two-way ANOVA, *F*_4,32_ = 20.45, *P* < 0.0001; **Figure 5B**), although no sex differences were detected (rostral IPSCs: *n* = 5; two-way ANOVA, *F*_1,32_ = 1.35, *P* > 0.1; caudal IPSCs: *n* = 5; two-way ANOVA, *F*_1,32_ = 1.15, *P* > 0.1; **Figure 5**).

**FIGURE 6:**
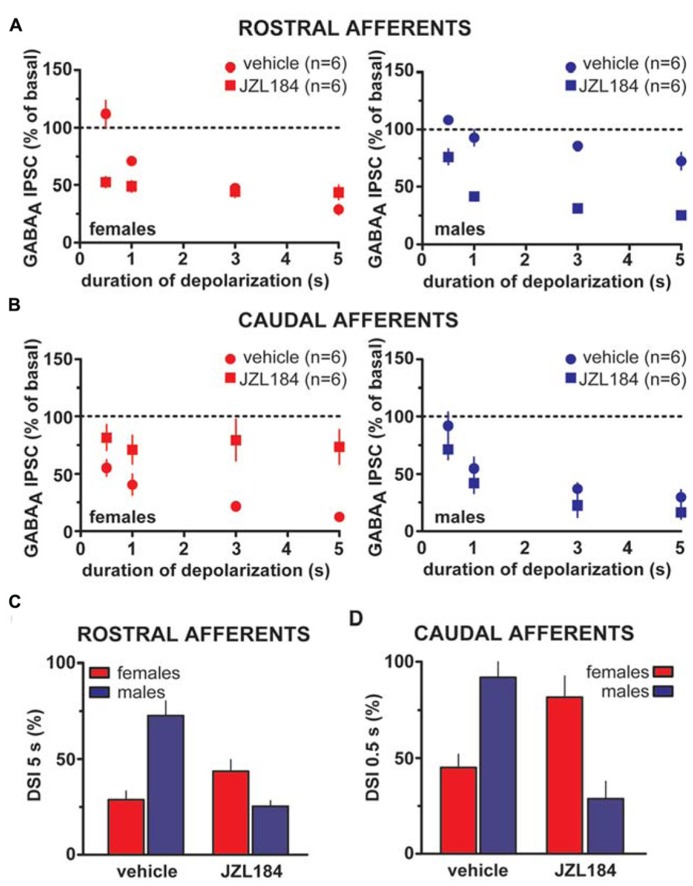
**Effect of MAGL inactivation on strength of DSI in male and female LH rats.** **(A)** The relationship between the depolarizing pulse duration and the relative amplitude of rostral GABA_A_ IPSCs obtained after 5–15 s after the end of depolarization in the presence (square symbols) and absence (circles) of MAGL inhibitor JZL184 in female (left panel) and male (right panel) LH rats is plotted. GABA_A_ IPSCs amplitude was normalized to the averaged value (dotted line) before depolarization. Each symbol represents the averaged value obtained from different cells. **(B)** The relationship between the depolarizing pulse duration and the relative amplitude of caudal GABA_A_ IPSCs obtained after 5–15 s after the end of depolarization in the presence (square symbols) and absence (circles) of MAGL inhibitor JZL184 in female (left panel) and male (right panel) LH rats is plotted. GABA_A_ IPSCs amplitude was normalized to the averaged value (dotted line) before depolarization. Each symbol represents the averaged value obtained from different cells. **(C,D)** Bar graphs summarizing the enhancing effect of JZL184 on DSI magnitude at both rostral (5 s; **C**) and caudal (0.5 s; **D**) afferents onto DA cells in male LH rats. Note that DSI at both inhibitory afferents in males is no longer different from DSI in females as measured during vehicle bath application. Data are expressed as mean ± SEM.

**FIGURE 7:**
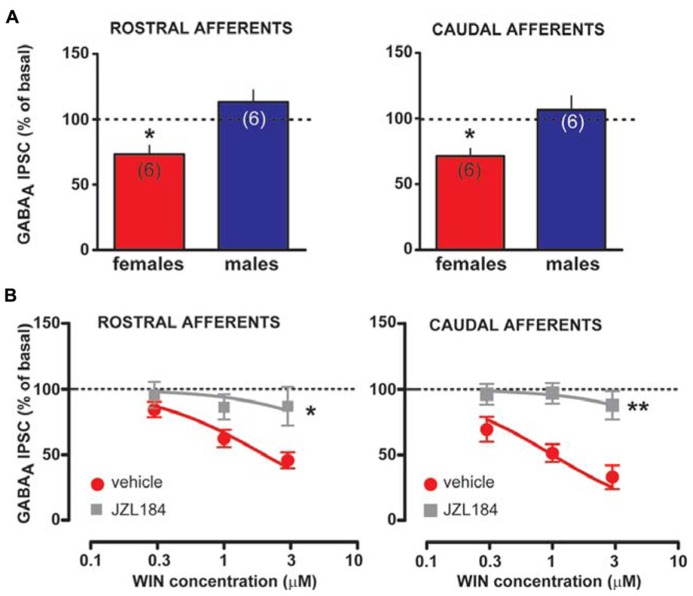
**Effect of MAGL inactivation on CB1 receptor function in female LH rats.** **(A)** Bar graphs showing the effect of MAGL inhibitor JZL184 on the amplitude of rostral (left panel) and caudal (right panel) GABA_A_ IPSCs in female and male LH rats is plotted. GABA_A_ IPSCs amplitude was normalized to the averaged value (dotted line) before JZL184/vehicle bath application. **(B)** Dose–response curves for percentage inhibition in GABA_A_ IPSC amplitude, which were recorded in female LH rats from VTA DA cells and evoked by stimulating either rostral (left panel, *n* = 6 for both sexes) or caudal (right panel, *n* = 6 for both sexes) afferents, produced by WIN in the presence (gray squares) or absence (red circles) of JZL184. Each symbol represents the averaged value ( ± SEM) obtained from different cells. GABA_A_ IPSCs amplitude was normalized to the averaged value (dotted line) before drug application. *P < 0.05, **P < 0.01.

Taken altogether, these results suggest that sex dimorphisms in rDSI and cDSI cannot be ascribed to sex specific differences in CB1 receptor number/function. Monoacylglycerol lipase (MAGL), the principal degradative enzyme for 2-AG (Blankman et al., 2007), is the rate-limiting step determining the time course of 2-AG-dependent DSE/DSI (Pan et al., 2009). We, next, examined the effects of the potent MAGL inhibitor JZL184 (Long et al., 2009) on rDSI and cDSI in both sexes. Bath application of JZL184 (100 nM) enhanced rDSI in both females (*n* = 6; two-way ANOVA, *F*_1__,3__3_ = 12.33, *P* < 0.01; **Figure 6A**, left panel) and males (*n* = 6; two-way ANOVA, *F*_1,33_ = 90.78, *P* < 0.0001; **Figure 6A**, right panel). In contrast, JZL184 affected cDSI in females (*n* = 6; two-way ANOVA, *F*_1,30_ = 10.09, *P* < 0.01; **Figure 6B**, left panel) but not in males (*n* = 6; two-way ANOVA, *F*_1,30_ = 2.62, *P* > 0.1; **Figure 6B**, right panel). Remarkably, both rDSI (5 s) and cDSI (0.5 s) were no longer different when male rat midbrain slices were treated with JZL184 (rDSI: unpaired *t*-test, *t* = 0.91, *P* > 0.1; cDSI: unpaired *t*-test, *t* = 1.43, *P* > 0.1; **Figures 6C,D)**. Noteworthy, in the presence of JZL184 both rDSI and cDSI were saturated in female LH rats, being the effect no longer dependent upon duration of depolarization (**Figures 6A,B**). Additionally, JZL184 was found to decrease *per se* GABA_A_ IPSC amplitude (~25%, paired *t*-test, *P* < 0.05 at both inputs), an effect that was sex specific (rostral IPSC: unpaired *t*-test, *t* = 2.9, *P* < 0.05; caudal IPSC: unpaired *t*-test, *t* = 3.6, *P* < 0.01; **Figure 7A**). The sex specific reduction of IPSCs and saturation/attenuation of DSI by acute JZL184 treatment at both sets of synapses could, therefore, be consistent with desensitization of CB1 receptors (Schlosburg et al., 2010). To test this hypothesis, we bath applied WIN (0.3–3 μM, 5 min) on JZL184-treated female rat slices, and found that WIN (1 and 3 μM) induced no depression of IPSCs in female JZL184-treated rat slices when compared with vehicle-treated counterparts (**Figure 7B**). In fact, the effect of WIN on GABA_A_ IPSCs recorded from VTA DA neurons, evoked by stimulating either rostral or caudal afferents, was blunted in JZL184–treated female rats (rostral IPSC: *n* = 6; two-way ANOVA, *F*_1,20_ = 6.70, *P* < 0.05; **Figure 7B**, left panel; caudal IPSC: *n* = 6; two-way ANOVA, *F*_1,2__0_ = 40.43, *P* < 0.0001; **Figure 7B**, right panel).

## DISCUSSION

Our findings reveal a tonic 2-AG signaling, which modulates inhibitory afferents impinging upon VTA DA neurons in female LH rats. 2-AG by differently depressing inhibitory synapses in a sex specific fashion might indirectly alter DA neuron functional state, and enhance their responsiveness towards excitatory inputs evoked by external (rewarding) stimuli, such as those associated with drugs of abuse. Particularly, sex specific tonic 2-AG signaling at caudal synapses (i.e., RMTg→VTA) might decrease aversive signal encoded by RMTg GABA cells, and increase the net hedonic/salient yield encoded by DA neurons.

Our observations substantially support the key role played by 2-AG in regulation of different forms of synaptic plasticity in the VTA, thus providing a critical modulation of behaviorally relevant DA neuron output (Melis and Pistis, 2012). The present data provide further evidence that 2-AG is the mediator of DSE/DSI in the VTA (Melis and Pistis, 2007). Indeed, both cDSI and rDSI were prevented when DA cells were loaded with a *sn*-1-DAGL inhibitor (i.e., THL), and enhanced when 2-AG degradation was prevented by blocking MAGL (i.e., JZL184). Accordingly, as shown in many other brain regions (Pan et al., 2009; Schlosburg et al., 2010; Yoshida et al., 2011; Zhong et al., 2011), 2-AG degradation by MAGL determines the strength of retrograde synaptic depression also in the VTA. Remarkably, when 2-AG clearance was decreased in DA cells recorded from male LH rats, DSI at both sets of inhibitory synapses were alike to those expressed by DA neurons in female LH rats under basal conditions. Hence, a different sensitivity/activity of the MAGL in females might underlie tonic 2-AG levels at this synapse. In addition, the finding that acute treatment with JZL184 potentiated and disrupted DSI in male and female LH rat DA neurons, respectively, corroborates the notion that inactivation of MAGL might impair specific endocannabinoid-mediated forms of synaptic plasticity due to partial desensitization of CB1 receptors (Schlosburg et al., 2010; Zhong et al., 2011). Indeed, we observed a sex specific persistent activation of CB1 receptors by 2-AG when MAGL was inactivated, which resulted in subsequent CB1 receptor desensitization detectable as either an attenuation/saturation of DSI or a blunted response to effective doses of CB1 receptor agonist WIN. CB1 receptor desensitization might also be a plausible explanation for the ineffectiveness of WIN to enhance VTA DA cell activity *in vivo* in female LH rats. If so, this would suggest a common target with 2-AG retrograde signal at this synapse. Nonetheless, sex-dependent changes in CB1 expression and function following CB1 receptor activation have already been described (Rubino et al., 2008; Burston et al., 2010). Conversely, no sex-dependent changes were observed in 2-AG brain content in both limbic and midbrain regions (Bradshaw et al., 2006; Llorente et al., 2008; Malinen et al., 2009), although these levels have previously been demonstrated to fluctuate as a function of the phases of estrous cycle (Bradshaw et al., 2006). It is worth to mention, however, that whether or not the production and degradation of 2-AG is under control and/or regulation of sex hormones has not been elucidated yet. Indeed, sex dimorphisms in both density and affinity of CB1 receptors have been reported to occur within the rodent midbrain (Rodriguez de Fonseca et al., 1994). Notably, CB1 receptor density and affinity were affected by ovary ablation and hormone-replacement, thus suggesting that sex steroid hormones might influence neuronal processes within the midbrain (Rodriguez de Fonseca et al., 1994).

Additionally, JZL184-induced inactivation of MAGL decreased GABA IPSCs amplitude at both rostral and caudal synapses impinging upon VTA DA neurons in female LH rats under basal conditions (i.e., 0.1 Hz stimulation, *V*_m_ = -70 mV). This scenario, being sex specific, is suggestive of tonic 2-AG levels acting on CB1 receptors. Alternatively, sex-dependent differences in maximal inhibition of MAGL activity might account for JZL184-induced effects. However, under similar conditions (i.e., juvenile rat brain) maximal inhibition of MAGL activity by JZL184 was found to be similar in female and male Sprague Dawley rats (Carr et al., 2011). However, whether or not this sex dimorphism is ascribed to differences in either make up, sensitivity and/or activity of MAGL in LH rats remains to be elucidated yet. Nonetheless, our findings support and extend previous studies showing that no sex differences in CB1 receptor expression/function do exist in the VTA of LH rats (Castelli et al., 2013), and that sex dimorphism in endocannabinoid modulation of synaptic function does exist in other brain regions such as the hippocampus (Huang and Woolley, 2012).

Deficient clearance of 2-AG in female LH rats, being larger at caudal vs rostral synapses, might enhance 2-AG half-life, and ultimately result in a targeted silencing of RMTg→VTA vs VP/NAcc→VTA synapses. A possible explanation for this phenomenon might be a sex-dependent difference in Km of MAGL. Hence, if Km is lower in males than females, only small amounts of 2-AG would be required for MAGL to be saturated in male LH rats. Consequently, MAGL maximum velocity would be reached at relatively low 2-AG concentrations in males when compared to female rats. Noteworthy, we observed that the shortest duration of depolarization is effective in reducing GABA IPSCs at caudal, but not rostral, synapses in female LH rats. This is particularly relevant given that this could be within the range of those occurring with frequency-summation of action potentials *in vivo*. Accordingly, VTA DA neurons in female LH rats under basal conditions (i.e., 0.1 Hz stimulation, *V*_m_ = -70 mV) display a paired-pulse facilitation at caudal but not rostral inhibitory synapses, suggestive of a decreased probability of GABA release from RMTg→VTA DA cells. This unique molecular convergence of 2-AG signaling at this given synapse might confer female LH rats less sensitivity to aversive properties of cannabinoids, crucial in the acquisition of self-administration behavior (Stolerman, 1992), and ultimately explain their propensity to acquire such a behavior (Fattore et al., 2007). Accordingly, *in vivo* VTA DA neurons in female LH rats appear to be subjected to a decreased RMTg influence when compared to their male counterparts. Also, their responses to *in vivo* CB1 receptor activation are blunted with respect to those observed in male rats (present study and French et al., 1997; Gessa et al., 1998). However, at this stage we have no explanation other than those above mentioned (e.g., CB1 receptor desensitization) for this phenomenon.

Importantly, VTA DA neurons primarily signal reward and salience (Lammel et al., 2008, 2012; Bromberg-Martin et al., 2010) to assign emotional/motivational valence to both external and internal stimuli, and endocannabinoids play an important role in fine tuning DA output via modulation of circuits in series (Melis et al., 2012). Further elucidation of both sex specific detailed synaptic connectivity of inhibitory inputs onto VTA DA cells, and sex-dependent enrichment with key molecular players required for 2-AG signaling machinery is required. Additionally, whether or not sex-specific tonic 2-AG signal sets different thresholds for gating subsequent synaptic plasticity, and ultimately alters postsynaptic cell excitability has to be established yet. Nonetheless, it is tempting to speculate that 2-AG-mediated DSI at these synapses might enable learning (i.e., motivational) processes, which are typically tightly controlled by an experience-based balance between appetitive and aversive properties. Remarkably, whether this sex dimorphism does exist in other brain areas has to be investigated yet, being the majority of the studies carried out in males. Nonetheless, while not all of sexually dimorphic effects of (exogenous) cannabinoids can be ascribed to sex differences in 2-AG signaling at these synapses, our results might contribute to unveil neurobiological mechanisms underlying sex disparity in vulnerability to cannabis dependence.

## Conflict of Interest Statement

The authors declare that the research was conducted in the absence of any commercial or financial relationships that could be construed as a potential conflict of interest.
